# The effect of *COMT Val158Met* and *DRD2 C957T* polymorphisms on executive function and the impact of early life stress

**DOI:** 10.1002/brb3.695

**Published:** 2017-04-12

**Authors:** Kristel Klaus, Kevin Butler, Simon J. Durrant, Manir Ali, Chris F. Inglehearn, Timothy L. Hodgson, Humberto Gutierrez, Kyla Pennington

**Affiliations:** ^1^ School of PsychologyUniversity of LincolnLincolnUK; ^2^ Section of Ophthalmology & NeuroscienceLeeds Institute of Biomedical SciencesSt James’ HospitalUniversity of LeedsLeedsUK; ^3^ School of Life SciencesUniversity of LincolnLincolnUK

**Keywords:** C957T polymorphism, catechol‐O‐methyltransferase, childhood trauma, *COMT*, dopamine receptor D2, *DRD2*, early life stress, executive function, Val158Met polymorphism

## Abstract

**Introduction:**

Previous research has indicated that variation in genes encoding catechol‐O‐methyltransferase (*COMT*) and dopamine receptor D2 (*DRD2*) may influence cognitive function and that this may confer vulnerability to the development of mental health disorders such as schizophrenia. However, increasing evidence suggests environmental factors such as early life stress may interact with genetic variants in affecting these cognitive outcomes. This study investigated the effect of *COMT Val158Met* and *DRD2 C957T* polymorphisms on executive function and the impact of early life stress in healthy adults.

**Methods:**

One hundred and twenty‐two healthy adult males (mean age 35.2 years, range 21–63) were enrolled in the study. Cognitive function was assessed using Cambridge Neuropsychological Test Automated Battery and early life stress was assessed using the Childhood Traumatic Events Scale (Pennebaker & Susman, 1988).

**Results:**

*DRD2 C957T* was significantly associated with executive function, with CC homozygotes having significantly reduced performance in spatial working memory and spatial planning. A significant genotype–trauma interaction was found in Rapid Visual Information Processing test, a measure of sustained attention, with CC carriers who had experienced early life stress exhibiting impaired performance compared to the CC carriers without early life stressful experiences. There were no significant findings for *COMT Val158Met*.

**Conclusions:**

This study supports previous findings that *DRD2 C957T* significantly affects performance on executive function related tasks in healthy individuals and shows for the first time that some of these effects may be mediated through the impact of childhood traumatic events. Future work should aim to clarify further the effect of stress on neuronal systems that are known to be vulnerable in mental health disorders and more specifically what the impact of this might be on cognitive function.

## 1 Introduction

Ever since the mapping of the human genome, identification of genetic determinants of complex psychiatric disorders such as schizophrenia, major depression or bipolar disorder has proven challenging (Allen et al., 2008). Indeed, genome‐wide association studies (GWAS) have shown that these disorders are highly polygenic (Barnett & Smoller, 2009; Schizophrenia Working Group of the Psychiatric Genomics, 2014; Shyn et al., 2011) with many genes exerting their influence on numerous neural pathways involved in complex aspects of brain function and likely being impacted upon by environmental events. It has been proposed that individual vulnerability and sensitization to the later development of mental health disorders may partly be regulated through variation in genes encoding proteins that impact on neurotransmitter and hormone systems such as those involved in dopamine, serotonin, and cortisol function (Caspi & Moffitt, 2006; Collip, Myin‐Germeys, & Van Os, 2008; van Winkel, Stefanis, & Myin‐Germeys, 2008; van Winkel, van Nierop, Myin‐Germeys, & van Os, 2013). Cognitive dysfunction is present in psychiatric disorders such as bipolar disorder (Bora, Yucel, & Pantelis, 2009) and major depression (Lau & Eley, 2010), and is considered a core symptom in schizophrenia (Young & Geyer, 2015), with pronounced impairments in general IQ as well as more specific prefrontal cortex‐mediated executive functions (Joyce et al., 2002). Cognitive dysfunction has also been observed in close relatives of schizophrenic (Snitz, MacDonald, & Carter, 2006) and bipolar disorder patients (Bora et al., 2009), further supporting the notion of a genetic component. Understanding how certain genetic vulnerabilities might affect or impair cognitive processes mediated by the prefrontal cortex (PFC) is of great importance given the functional abnormalities of the PFC present in schizophrenia and bipolar disorder (Pennington et al., 2008). Furthermore, the PFC appears to be particularly susceptible to early life stress. This susceptibility may be due to its high density of glucocorticoid receptors and dopaminergic projections (Pani, Porcella, & Gessa, 2000), and the fact that it is one of the last brain regions to reach maturity (Gogtay & Thompson, 2010; Gogtay et al., 2004). In support of this, deficits in functions mediated by PFC and cortico‐striatal networks, such as attention, working memory and other executive functions, have also been reported in individuals who have experienced childhood trauma (Bos, Fox, Zeanah, & Nelson, 2009; Colvert et al., 2008).

The PFC receives key ascending dopaminergic inputs from the midbrain and other subcortical areas (Kellendonk et al., 2006; Tekin & Cummings, 2002), and emerging evidence suggests the existence of an optimal level of dopamine needed for efficient PFC functioning (Goldman‐Rakic, Muly, & Williams, 2000). Two genes that have been associated with various psychiatric and neurological disorders and which have attracted attention due to their role in cognitive and emotional processes are the genes encoding for the enzyme catechol‐O‐methyltransferase (*COMT*; Dickinson & Elvevåg, 2009; Scheggia, Sannino, Luisa Scattoni, & Papaleo, 2012; Tunbridge, Harrison, & Weinberger, 2006) and the dopamine receptor D2 (*DRD2*; Frank & Fossella, 2011; Huertas, Bühler, Echeverry‐Alzate, Giménez, & López‐Moreno, 2012; Kellendonk et al., 2006). *COMT* is responsible for breaking down ~50–60% of dopamine produced in the frontal cortex (Yavich, Forsberg, Karayiorgou, Gogos, & Männistö, 2007). The *Val158Met* (rs4680) polymorphism is the most common variation in the *COMT* gene (Tunbridge et al., 2006) whereby a single guanine to arginine (G/A) base pair substitution at codon 158 results in a valine (Val) to methionine (Met) substitution, which leads to ~40% lower enzyme activity in the Met carriers (Chen et al., 2004). Research investigating the association between genetic variation in *COMT Val158Met* and executive function, attention, and working memory in healthy people as well as schizophrenic patients has had mixed findings (Barnett, Jones, Robbins, & Müller, 2007; Barnett, Scoriels, & Munafò, 2008). It is possible that the presence of adverse life events may mediate some of the effects of this *COMT* variant on cognition. Indeed, recent studies have shown that the Met allele is associated with greater stress responsivity and hypothalamic‐pituitary–adrenal axis activity (Hernaus et al., 2013; Walder et al., 2010), with impaired working memory function under acute stress (Buckert, Kudielka, Reuter, & Fiebach, 2012) and with poorer affective decision‐making under chronic stress conditions in healthy subjects (He et al., 2012). Furthermore, mice with the *COMT* gene removed exhibited decreased resilience to stress, but better working memory performance whereas overexpression of the human *COMT* Val polymorphism resulted in a blunted stress response and impaired attentional set‐shifting ability, as well as reduced working and recognition memory (Papaleo et al., 2008). More recently, it was found that Val/Val homozygotes with a diagnosis of schizophrenia spectrum disorder performed worse on an executive function task than Met carriers in the absence of childhood adversity, but, surprisingly, the Val/Val homozygotes with a history of physical abuse performed better than Met carriers on the same task (Green et al., 2014), suggesting a developmental increase in dopamine activity in the PFC in the context of trauma.

Dopamine D2 receptors and the genetic polymorphisms encoding for these receptors have also been associated with various aspects of cognition. D2 receptor availability in the caudate has been associated with performance on executive function tests requiring mental flexibility, abstraction, response inhibition and attention in healthy individuals (Volkow et al., 1998) and D2 receptor occupancy has been associated with vigilance and neurocognitive function in schizophrenia patients (Sakurai et al., 2012), whereas the blockade of D2 receptors by risperidone correlates with attentional deficits in schizophrenia (Uchida et al., 2009). Animal studies have shown that mutant mice lacking D2 receptors exhibit impaired spatial working memory and attentional abilities (Glickstein, DeSteno, Hof, & Schmauss, 2005; Glickstein, Hof, & Schmauss, 2002) and over‐expression of D2 receptors in striatum has similarly been associated with impaired working memory performance in mice (Kellendonk et al., 2006). The *C957T* (rs6277) polymorphism in the D2 receptor has recently gained interest due to its proposed role in the development of schizophrenia. More specifically, the C allele has been associated with heightened schizophrenia risk in Caucasian samples (Liu et al., 2014), whereas the T allele has been found to confer risk toward schizophrenia in Asian populations (Fan et al., 2010). While the *C957T* mutation leads to a synonymous substitution, it has been shown that this variant affects D2 receptor availability in both striatal (Hirvonen et al., 2004, 2005; Hirvonen, Laakso, et al., 2009) and cortical regions (Hirvonen, Lumme, et al., 2009), with CC homozygosity being associated with lower striatal D2 receptor availability (CC < CT < TT; Hirvonen et al., 2005, 2004; Hirvonen, Laakso, et al., 2009), but with higher D2 receptor availability in the cortex and thalamus (CC > CT > TT; Hirvonen, Lumme, et al., 2009). Consequently, it has been suggested that this single‐nucleotide polymorphism (SNP) may play a role in the abnormal mesocortical and mesolimbic dopamine transmission evident in schizophrenia (Fan et al., 2010).

The genetic studies looking at the association of *DRD2 C957T* with cognitive function have generally shown that CC homozygosity is associated with poorer performance on tests of executive function and working memory in the general population (Rodriguez‐Jimenez et al., 2006; Xu et al., 2007), decreased general cognitive ability in elderly healthy males and females (Bolton et al., 2010), impaired executive function and cognitive flexibility in HIV‐infected individuals who abuse alcohol (Villalba, Devieux, Rosenberg, & Cadet, 2015), and poorer performance in an attentional switching task in CC homozygotic females (Gurvich & Rossell, 2015). The T allele and TT homozygosity has been associated with better avoidance learning from negative outcomes in both forced choice and reaction time tasks (Frank, Doll, Oas‐Terpstra, & Moreno, 2009; Frank, Moustafa, Haughey, Curran, & Hutchison, 2007), better striatally mediated reflexive learning (Xie, Maddox, McGeary, & Chandrasekaran, 2015) and rule‐based category learning in healthy young adults (Byrne, Davis, & Worthy, 2016), and with superior performance in an attentional switching task in TT homozygotic males (Gurvich & Rossell, 2015). On the other hand, CC homozygotes show better performance in a backward serial recall task (Li et al., 2013), and the increasing number of C alleles has been associated with better performance in tasks of vocabulary, verbal fluency and digit span among those with clinical and familial risk of psychosis, but not among the control group (Ramsay et al., 2015). Others have found increased reaction time variability in a response inhibition task in CT heterozygotes (Gurvich & Rossell, 2014). Prefrontal cortical D2 receptors are also proposed to affect responsivity to stress via subcortical projections by attenuating dopamine release in the midbrain via a negative feedback system (Deutch, 1993).

### 1.1 Aims and hypotheses

Although previous attempts have been made to investigate the role of gene variants involved in dopaminergic functioning on cognitive performance and the potential role of stressful life events, studies are in their infancy. No study as far as the authors are aware has considered the potential interactive effects of *DRD2 C957T* and *COMT Val158Met* with early life stress in performance on a comprehensive cognitive battery of executive function tests (working memory, attention, strategy use, planning, and decision‐making) in healthy adults. Consequently, this study aimed to investigate whether healthy adult males with either Met homozygosity of *COMT* or carriers of T allele of *DRD2* will show better cognitive performance. In addition, based on previous findings we aimed to investigate whether individuals with higher levels of early life stress will have more impaired cognitive function if they are homozygous for the Met allele at *Val158Met* or the C allele at *C957T*.

## 2 Materials and Methods

### 2.1 Participants

One hundred and twenty‐two healthy adult males [mean age 35.15 years (*SD *= 11.02), range 21–63 years] were recruited from in and around Lincoln, UK as part of a larger ongoing programme of research. The majority of the participants were from Caucasian ethnicity (115 individuals), with the remaining participants being of mixed race origin (2), Asian/Asian British (2), Iranian/British Iranian (2), and Arab (1) ethnicity. Individuals with a self‐reported current diagnosis of a major psychiatric disorder, antipsychotic medication prescription or current drug or alcohol addiction problems were excluded from taking part in the study at the point of first contact. All participants were additionally assessed for symptoms of psychopathology using the Hospital Anxiety and Depression Scale (HADS; Zigmond & Snaith, 1983) and for current stress levels using a 14‐item Perceived Stress Scale (PSS‐14; Cohen, Kamarck, & Mermelstein, 1983). Eighty‐six per cent of the participants were within the normal or mild range on the anxiety subscale and 99% were within the normal to mild range on depression subscale. Ninety per cent of the participants had the PSS‐14 score within the range of 0–28, previously defined as “no stress” range (Amr, El Gilany, & El‐Hawary, 2008; Shah, Hasan, Malik, & Sreeramareddy, 2010). The participants who scored above the mild anxiety levels on HADS and above “no stress” range on PSS‐14 were not disproportionately distributed among genotype groups. All participants gave written informed consent before being tested. The study was approved by the School of Psychology Research Ethics Committee at the University of Lincoln.

### 2.2 Neurocognitive tests

Each participant carried out a set of cognitive tests using The Cambridge Neuropsychological Test Automated Battery (CANTAB; Cambridge Cognition, Cambridge, UK) which applies touch‐screen technology, enabling participants to be tested in a standardized manner. The cognitive tests were always administered in the same order and took approximately 1 h to complete. The test battery consisted of one training/quality control test (Motor Screening Task [MOT]), one motor test (Reaction Time [RTI]) and four tests assessing cognitive processes associated with PFC and executive function (Spatial Working Memory [SWM]; Rapid Visual Information Processing [RVP]; One Touch Stockings of Cambridge [OTS] and Intra‐Extradimensional Set Shift [IED]). Full details about these are available at http://www.cambridgecognition.com.

### 2.3 Measurement of early life stress

Stressful early life events were assessed using the Childhood Traumatic Events Scale (CTES; Pennebaker & Susman, 1988), which asks individuals retrospectively about the occurrence of six categories of trauma: death of a close friend or relative, parental separation or divorce, traumatic sexual experience, physical violence, major illnesses or injuries, or other traumatic experiences prior to the age of 17. The scale also asks the individual to rate the severity of the traumatic event and the extent that the person remembers confiding in others about it, both responses being rated on a scale from 1 to 7, where 7 is extremely traumatic and with substantial beliefs that they confided in others at the time of the event. Participants had to rate the event as 6 or 7 in order for it to be classified as a traumatic event.

### 2.4 Procedure

All test administrators were trained to use CANTAB in a standardized manner and instructions were given to the participants according to the test administration guide from Cambridge Cognition (http://www.cambridgecognition.com). Eighty‐six per cent of the participants were tested in the morning. The participants provided a saliva sample for genetic analysis using the Oragene (OG‐500; DNA Genotek Inc., Ottawa, ON, Canada) collection kit. This was collected after completing MOT, RTI, SWM, and RVP in order to allow a break in the testing session and prevent mental fatigue (Trejo, Kubitz, Rosipal, Kochavi, & Montgomery, 2015). As this was part of a wider programme of research, after cognitive testing, the participants were asked to fill in a series of questionnaires about demographic information, medical history and current medication status, substance use history, current alcohol and drug use, mood status, current perceived stress, and childhood adversity.

### 2.5 *COMT* Sequencing

DNA was extracted from saliva using Oragene prepIT L2P (DNA Genotek Inc. http://www.dnagenotek.com) according to the manufacturer's protocol. The resulting DNA was diluted to 100–1000 ng/μl, using 1× Tris‐EDTA (TE) buffer (Cat #: 93302; Sigma‐Aldrich, Dorset, UK) and assessed for purity and concentration using a NanoDrop™ ND1000 Spectrophotometer (Thermo Fisher Scientific Inc., Hemel Hempstead, Hertfordshire, UK). Primers were designed using the online software Primer3 v.0.4.0 (http://frodo.wi.mit.edu/). Polymerase chain reaction (PCR) on Techne TC‐5000 Thermal Cycler (Bibby Scientific Ltd., Staffordshire, UK) generated a 220‐bp product, using the primers COMT‐F1 (GGG CCT ACT GTG GCT ACT CA) and COMT‐R1 (GGG TTT TCA GTG AAC GTG GT). The resulting fragment contains the rs4680 SNP located in exon 4 of the *COMT* gene (Genbank accession number Z26491). Briefly, each 10 μl reaction consisted of 100–1000 ng of human genomic DNA, 1.6 pmol/L of the forward and reverse primers, 100 μmol/L dNTPs, 10× PCR Amplification buffer (Cat #: 11495–017, Invitrogen, Paisley, UK), 0.1 unit of Taq DNA polymerase, 1 mol/L Betaine and 4% DMSO. An initial denaturation step of 95°C for 2 min was followed by 40 cycles of 94°C for 30 s, 55°C for 45 s, and 72°C for 45 s. A final extension of 72°C for 5 min completed the session. PCR products were electrophoresed on a 1.5 % agarose gel stained with ethidium bromide and visualized using the ultraviolet light filter on the ChemiDoc Imaging system (BioRad Laboratories Ltd., Hemel Hempstead, Hertfordshire).

PCR products were digested with ExoSAP‐IT (Cat #: 78201; Affymetrix USB, Santa Carla, CA, USA) and sequencing reactions were carried out using the Big Dye Terminator v3.1 Cycle Sequencing Kit (Cat #: 4337457; Applied Biosystems, Warrington, UK). These products were run on an ABI3130xl Genetic Analyzer (Applied Biosystems). The sequencing primer was COMT‐F1. Chromatograms were viewed using Chromas Lite version 2.1 (Technelysium Pty Ltd, South Brisbane, Qld, Australia) and verified by two independent researchers.

### 2.6 *DRD2* Genotyping

Genotyping for *DRD2* rs6277 was carried out according to the manufacturer's instructions using TaqMan^®^ Pre‐Designed SNP Genotyping Assays technology with the predesigned SNP assay containing locus‐specific primers and fluorogenic allele‐specific probes for rs6277 (Identification no.: C__11339240_10) and Master Mix (Cat #: 4371353). All reagents and equipment were ordered from Applied Biosystems unless otherwise specified. The 25 μl reaction volume contained 30 ng of genomic DNA, 0.66 μl of 40× assay mix, 12.50 μl of TaqMan^®^ Universal PCR Master Mix, and 11.25 μl of DNAse‐free water (Cat #: 4502; Sigma‐Aldrich). Amplification was carried out using ABI StepOne™Plus Real‐Time PCR System with 96‐well plates. The thermal profile was 60°C for 30 s, 95°C for 10 min, followed by 50 cycles at 92°C for 15 s and 60°C for 1 min. PCR software (StepOnePlus™ v2.0) measured SNP‐specific fluorescence and genotyped each sample post‐PCR.

### 2.7 Statistical analysis

A priori power calculations were carried out using G*Power version 3.1.9.2 (Faul, Erdfelder, Lang, & Buchner, 2007), which suggested that *N *=* *127 would be needed for detecting medium effect sizes (*f *=* *.25) with a power of 0.70. Post hoc power analysis based on the current sample of *N *=* *122 suggested an actual power of 0.68 to detect medium effect size. All data were analyzed using SPSS version 21 (IBM Corp., Armonk, NY, USA). The main effects of genotype and childhood adversity, and their interaction, on cognitive measures were investigated using a two‐way analysis of covariance (ANCOVA). Dependent variables were the primary outcome measures suggested by CANTAB (Cambridge Cognition). If any of the primary outcome measures yielded significant results, secondary outcome measures were also investigated. Age and years of education were included as covariates in all ANCOVA analyses, and RTI median reaction time was additionally included as covariate in RVP analyses. These covariates were chosen due to their correlation with some or all of the cognitive test outcomes. Although ethnicity‐specific differences in the direction of the association between the *DRD2 C957T* alleles and schizophrenia have been reported (Liu et al., 2014), ethnicity was not associated with cognitive performance in this study and was therefore not included as a covariate in the analyses. Assumptions for an ANCOVA were checked and a Shapiro–Wilk test was used to determine whether the continuous dependent variables were normally distributed. If the assumption of normality was violated, the data were transformed (1/x) and/or outliers (± 3SD) were removed and normality tested once more. Where the ANCOVA results were significant, Bonferroni corrected *t* tests were used for post hoc pairwise comparisons. *p* value of .05 after multiple test corrections was used to determine significance in all statistical analyses.

## 3 Results

### 3.1 Genotypic distribution

*COMT Val158Met* genotype could not be clearly identified for one individual, so all results for *COMT* are based on 121 individuals, while the *DRD2 C957T* genotype results are based on 122 individuals. The genotype distribution conformed to the Hardy–Weinberg Equilibrium for both *COMT Val158Met* (χ^*2*^ **= 0.078, *df *= 1, *p *=* *.780) and *DRD2 C957T* (χ^*2*^ **= 0.066, *df *= 1, *p *=* *.797). Tables 1 and 2 show the genotype distributions and demographic information for the participants. One‐way ANOVA, Kruskal–Wallis and chi‐squared tests did not reveal any significant differences in demographic characteristics, HADS or PSS‐14 scores, nor traumatic events between genotypes, *p *>* *.05 in all cases.

**Table 1:** Demographic information, questionnaire scores, and traumatic events distributions for *COMT Val158Met* genotypes in all participants (*N *=* *121)

	Met Met	Met Val	Val Val	*F/*χ^*2*^	*p*
*N*	28	62	31		
Age *M (SD*)	35.79 (11.89)	35.48 (10.36)	33.69 (11.82)	1.30	.522
Education (years) *M* (*SD*)	14.11 (2.56)	14.27 (2.38)	15.29 (2.24)	2.352	.100
Ethnicity					
Caucasian	27	58	29		
Other	1	4	2		
HADS Anxiety *M* (*SD*)	5.75 (3.88)	6.53 (3.77)	6.58 (3.37)	1.645	.439
HADS Depression *M* (*SD*)	2.82 (2.53)	3.26 (2.66)	3.58 (2.88)	1.052	.591
PSS‐14 *M* (*SD*)	18.96 (8.50)	20.39 (6.98)	21.42 (8.86)	0.724	.487
NART *M* (*SD*)	110.65 (8.27)	111.87 (6.47)	110.83 (7.75)	0.331	.719
Trauma/No Trauma	13/15	26/36	7/24	4.379	.112

Means with standard deviations (*M, SD*), test statistic (*F/*χ^*2*^), and significance level (*p*) are provided for age, education and questionnaire scores for *COMT Val158Met* genotypes. Ethnicity and Trauma/No Trauma groups show the number of participants in each group. Trauma* *=  a rating of 6 or 7 on CTES trauma scale (Pennebaker & Susman, 1988); No Trauma* *=  a rating of <6 on CTES trauma scale or no traumatic event. HADS* *= Hospital Anxiety and Depression Scale; PSS‐14 =  Perceived Stress Scale 14; NART* *=  National Adult Reading Test, scores are presented for native English speakers only. HADS scores: 0–7 =  normal, 8–10 =  mild, 11–15 =  moderate, and ≥16 =  severe (Snaith & Zigmond, 1994). PSS‐14 scores: 0–28 =  low stress, 29–56 =  high stress (Amr et al., 2008; Shah et al., 2010).

**Table 2:** Demographic information, questionnaire scores, and traumatic events distributions for *DRD2 C957T* genotypes in all participants (*N *=* *122)

	TT	TC	CC	*F/*χ^*2*^	*p*
*n*	35	62	25		
Age *M* (*SD*)	35.80 (11.03)	34.24 (10.05)	36.47 (13.37)	0.640	.726
Education (years) *M* (*SD*)	14.94 (2.50)	14.32 (2.41)	14.12 (2.42)	1.027	.361
Ethnicity					
Caucasian	33	58	24		
Other	2	4	1		
HADS Anxiety *M* (*SD*)	6.37 (4.63)	6.55 (3.33)	6.16 (3.30)	0.556	.757
HADS Depression *M* (*SD*)	3.29 (3.13)	3.23 (2.67)	3.32 (2.04)	0.834	.659
PSS‐14 *M* (*SD*)	20.46 (8.56)	20.05 (7.80)	21.12 (7.06)	0.167	.847
NART *M* (*SD*)	112.30 (7.17)	110.54 (7.46)	111.72 (6.64)	0.647	.526
Trauma/No Trauma	15/20	25/37	6/19	2.576	.276

See Table 1 legend for explanation.

### 3.2 Effects of genotypic variation and childhood trauma on cognitive function

**Figure 1:**
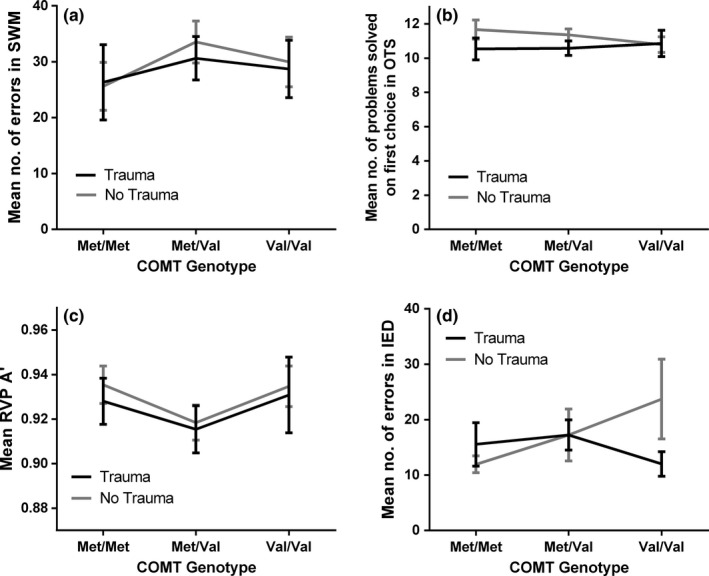
(a) Mean number of errors made in the Spatial Working Memory (SWM) test. (b) Mean number of problems solved on first choice in the One Touch Stockings of Cambridge (OTS) test. (c) Mean A’ score (sensitivity to the target, considering both hits and false alarms) in the Rapid Visual Information Processing (RVP) test. (d) Mean number of errors made in the Intra‐Extradimensional Set Shift (IED) test. All results are displayed across *COMT* genotypes, for both Trauma and No Trauma group separately. Trauma categories are based on Childhood Traumatic Events Scale (CTES; Pennebaker & Susman, 1988), where Trauma* *=  a rating of 6–7 on trauma scale and No Trauma* *=  a rating <6. Error bars show ±1 standard errors

A two‐way ANCOVA with *COMT Val158Met* genotype and childhood trauma category as fixed factors and age and education as covariates did not result in any significant effect on cognitive test outcomes, *p *>* *0.05 in all cases (see Figure 1a–d).

**Figure 2:**
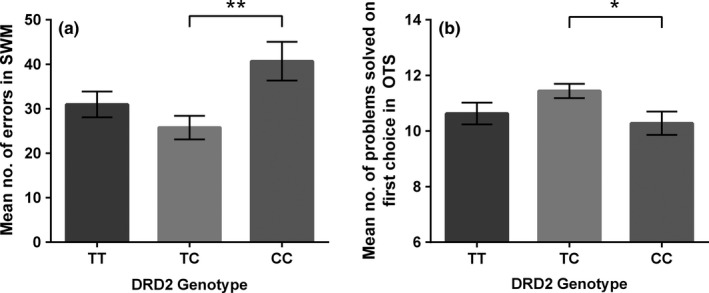
(a) Mean number of errors (±SEM) made in the Spatial Working Memory (SWM) test across *DRD2* genotypes. (b) Mean number of problems solved (±SEM) on first choice in the One Touch Stockings of Cambridge (OTS) test across genotypes. *p ≤ .05, **p ≤ .01

Results from two‐way ANCOVA with *DRD2 C957T* genotype and childhood trauma category as fixed factors and age and education as covariates showed that *DRD2 C957T* polymorphism had a significant main effect on the number of errors made in the SWM test (*F*_2,114_ **= 4.664, *p *=* *.011, partial η^2^ **= .076). Post hoc analyses revealed that this significant difference was in the responses given by the TC and CC genotypes, with CC homozygotes making significantly more errors than TC heterozygotes (*p *=* *.010) (see Figure 2a). *DRD2* genotype also had a near‐significant effect on strategy use in the SWM test (*F*_2,114_ **= 2.897, *p *=* *.061, partial η^*2*^ **= .048), with TC heterozygotes exhibiting better strategy use, as evidenced by starting the search with the same box more often than CC homozygotes (*p *=* *.069) (see Figure S1).

*DRD2* genotype also had a significant main effect on the number of problems solved on first choice in OTS (*F*_2,114_ **= 4.066, *p *=* *0.020, partial η^2^ **= .067). Post hoc analyses showed that TC heterozygotes solved significantly more problems on first choice than CC homozygotes (*p *=* *.049) (see Figure 2b). Two‐way ANCOVA on the secondary outcome measures showed that *DRD2* genotype had a significant main effect on the mean number of choices to correct answer in OTS (*F*_2,114_ **= 3.914, *p *=* *0.023, partial η^*2*^ **= .064). TC carriers took, on average, significantly fewer attempts to solve the problems than CC carriers (*p *=* *0.035) (see Figure S2).

**Figure 3:**
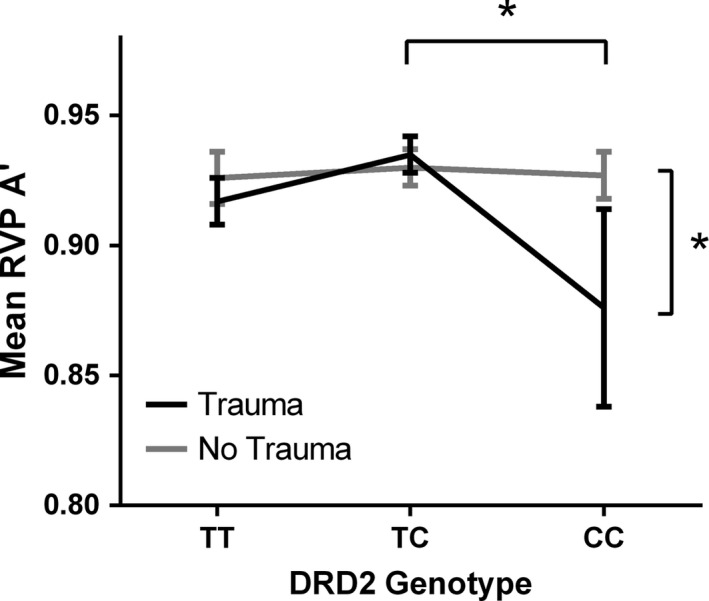
Mean A’ score (sensitivity to the target, considering both hits and false alarms) in the Rapid Visual Processing (RVP) task across *DRD2* genotypes, displayed for both Trauma and No Trauma group separately. Trauma categories are based on Childhood Traumatic Events Scale (CTES; Pennebaker & Susman, 1988), where Trauma* *=  a rating of 6–7 on trauma scale and No Trauma* *=  a rating <6. Error bars show ±1 standard errors. One outlier was removed from the *DRD2* ‐ Trauma Group interaction analysis due to a score of <3SD. *p ≤ .05

**Figure 4:**
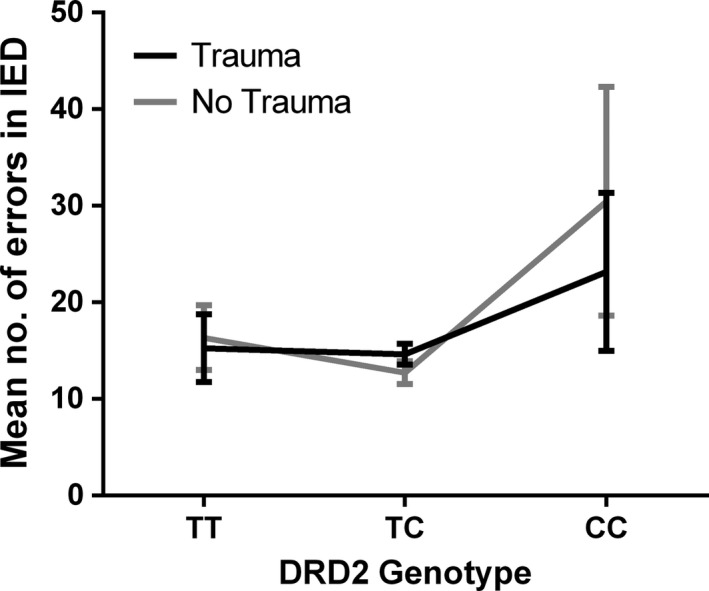
Mean number of errors made in the Intra‐Extradimensional Set Shift (IED) test across *DRD2* genotypes, displayed for both Trauma and No Trauma group separately. Trauma categories are based on Childhood Traumatic Events Scale (CTES; Pennebaker & Susman, 1988), where Trauma* *=  a rating of 6–7 on trauma scale and No Trauma* *=  a rating <6. Error bars show ±1 standard errors

We also identified a significant association between *DRD2 C957T* genotype, childhood trauma, and genotype–trauma group interaction on the measure of RVP A′(*F*_2,112_ **= 4.358, *p *=* *.015, partial η^*2*^ **= .072; *F*_1,112_ **= 4.672, *p *=* *.033, partial η^2^ **= .040; *F*_2,112_ **= 4.306, *p *=* *.018, partial η^*2*^ **= .069 respectively). Post hoc tests showed that the main effect of *DRD2* genotype arose due to the difference between TC and CC carriers, as TC heterozygotes were significantly more sensitive to target sequences, compared to CC homozygotes (*p *=* *.024), and the trauma group scored significantly lower on the measure of A’, compared to the No Trauma group. The genotype–childhood trauma interaction emerged from the difference in the CC homozygotes’ scores. Figure 3 shows that CC homozygotes who had experienced childhood trauma were significantly less sensitive to the target sequences, as measured by A’, compared to those who had not experienced childhood traumatic events. Analyses on the secondary RVP outcome measures revealed a significant main effect of *DRD2* genotype, a marginally significant main effect of childhood trauma, and a significant interaction between *DRD2* genotype and childhood trauma on the probability of hit in RVP (*F*_2,113_ **= 3.674, *p *=* *.028, partial η^*2*^ **= .061; *F*_1,113_ **= 3.789, *p *=* *.054, partial η^*2*^ **= .032; *F*_2,113_ **= 4.140, *p *=* *.018, partial η^*2*^ **= .068, respectively). TC heterozygotes were more likely to hit the target sequences than CC homozygotes at trend level (*p *=* *.057). The No Trauma group showed superior performance compared to the Trauma group. The interaction between the genotype and childhood trauma was particularly pronounced among the CC homozygotes, as CC homozygotes who had experienced childhood trauma were less likely to hit the target sequences than those without childhood traumatic events. There was also a significant *DRD2* polymorphism and childhood trauma interaction on the number of false alarms made in the RVP test (*F*_2,113_ **= 5.337, *p *=* *.006, partial η^*2*^ **= .086), with the difference again emerging from CC carriers. CC homozygotes from the Trauma category made more errors than those from No Trauma category (see Figure S3a and b). We did not detect any significant findings for the performance in the IED test, *p *>* *.05 (see Figure 4).

## 4 Discussion

This study aimed to investigate how two dopamine‐related gene variants, *COMT Val158Met* and *DRD2 C957T*, affect performance on a battery of executive function tests, and further to investigate whether genotype might modulate the effects of early life stress on cognitive outcomes in a sample of healthy males. In contrast to our initial hypothesis, we found no evidence that the *COMT Val158Met* polymorphism differentially affects executive function in the proposed genotype (Met homozygotes), nor did these findings change once we took into account the occurrence of early life stress. However, we confirmed our hypothesis that individuals homozygous for the C allele at *C957T* of the *DRD2* gene performed significantly poorer in tasks measuring different aspects of executive function and that experiencing early life stress leads to further impaired function. The negative finding for an effect on cognitive function in the *COMT Val158Met* SNP is in accordance with several other studies, including a meta‐analysis by Barnett et al. (2008), which concluded that *COMT Val158Met* has little if any effect on cognitive function. However, contrary to previous findings (Green et al., 2014; He et al., 2012), we failed to find an interaction between *COMT* and early life adversity on cognitive measures, a proposed mechanism by which variants in the *COMT* gene may contribute toward executive function performance. One possibility is that *COMT* only interacts with certain types of trauma. Indeed, Green et al. (2014) found that *COMT* interacted with physical abuse, but not with emotional abuse or emotional neglect, in affecting performance on an executive function test. Unfortunately, it was not possible to investigate the specific types of trauma and their interaction with genotype on cognition in the current study.

Variation in *DRD2 C957T* polymorphism was found to significantly affect performance in tests of spatial working memory (SWM), sustained attention (RVP) and spatial planning (OTS), with a particularly pronounced difference between CC homozygotes and CT heterozygotes. Overall, CC homozygotes made more errors and had poorer strategy use in SWM, compared to CT heterozygotes. CC homozygotes also showed least sensitivity to the target, with further analyses indicating that CC homozygotes had both a lower probability of hit and more false alarms in the RVP test, but only if childhood trauma category was also taken into account. Finally, CC homozygotes solved fewer problems on first choice in OTS and took on average more choices to achieve the correct answer, compared to CT heterozygotes. In general, these findings are in accord with previous studies that have reported CC homozygotes being compromised when compared to CT and TT carriers in working memory (Xu et al., 2007) and tests of executive function (Rodriguez‐Jimenez et al., 2006). CC genotype has been associated with lowest striatal D2 receptor availability and supposedly higher synaptic dopamine levels in the striatum, compared to other genotypes (Hirvonen et al., 2004, 2005; Hirvonen, Laakso, et al., 2009). Lower striatal D2 receptor availability has previously been associated with poorer executive function in healthy people (Cervenka, Bäckman, Cselényi, Halldin, & Farde, 2008; Reeves et al., 2005; Slagter et al., 2012; Volkow et al., 1998) and in individuals with a diagnosis of schizophrenia (Yang et al., 2004), therefore potentially explaining the poorer performance of CC homozygotes in our tests. The poorer performance of CC homozygotes in this and previous behavioral studies may lend evidence to the recessive C allele model. Indeed, the outcomes of the ANCOVA models remained largely unchanged by grouping TT and TC carriers (data not shown). However, other studies have found dose‐dependent effects of *DRD2 C957T* variant on both cognitive outcomes (e.g., Byrne et al., 2016) and on D2 receptor availability in both striatal (e.g., Hirvonen et al., 2005) and extrastriatal areas (e.g., Hirvonen, Lumme, et al., 2009), or even an inverted‐U‐shaped pattern in cognitive performance, whereby no significant difference in the performance between CC and TT genotypes was detected (Gurvich & Rossell, 2014). The absence of significant difference in the performance between CC and TT homozygotes in the current study may similarly suggest the inverted‐U‐shaped dopamine action, whereby intermediate levels of dopamine lead to superior cognitive performance (Cools & D'Esposito, 2011). Regardless of whether a recessive pattern or inverted‐U‐shaped pattern applies here, we believe that investigating genotype groups separately provides a more detailed picture of the nature of this gene variant — a model increasingly adopted by researchers in this area. Contrary to previous findings on the effect of *C957T* on WCST performance in Rodriguez‐Jimenez et al. (2006) study, we did not detect an effect of *C957T* in performance on the IED task, a computerized analog of WCST.

In the test of sustained attention, we found main effects of *DRD2* genotype and childhood trauma, as well as a genotype–trauma interaction on the primary outcome measure of A’, and the secondary outcome measures of probability of hit and the number of false alarms. There were no interactions of genotype with current stress or anxiety levels on cognitive outcomes in our study, as measured by the PSS‐14 and HADS, suggesting that this effect is restricted to the occurrence of early life stress as measured by the CTES. To the best of our knowledge, this is the first report of such interaction between *C957T* and early life stress on cognitive functioning. Although the decreased D2 receptor binding (CC genotype) has been associated with poorer performance on timed attention tests in previous studies (Slagter et al., 2012; Yang et al., 2004), the main effect of genotype in our study emerged in two‐way ANCOVA with childhood trauma category as another fixed factor, showing that CC homozygotes who have experienced childhood trauma perform more poorly on a sustained attention test, compared to those who have not experienced childhood adversity. The effect of early life stress on cognitive outcomes has been repeatedly demonstrated in earlier studies (Bos et al., 2009; Colvert et al., 2008), and it is to be expected due to the long maturation period of the PFC (Gogtay & Thompson, 2010; Gogtay et al., 2004) and high concentration of glucocorticoid receptors and dopaminergic projections in the PFC (Pani et al., 2000). However, it should be noted that the genotype–early life stress interaction was only found in the RVP test. Although RVP is primarily a test of sustained attention, the significant findings within this test were strongly driven by the mean number of false alarms, an indicator of response inhibition (Joos et al., 2013), the latter considered as one of the key components of executive function (Diamond, 2013). It could therefore be hypothesized that processes relating to response inhibition are particularly vulnerable to stress in key stages of development. Performance on inhibitory control tests has been associated with both D2 receptor availability and childhood trauma in previous studies (Ghahremani et al., 2012; Marshall et al., 2016) but the more precise effect of stress on the functioning of cortico‐striatal circuitry and on cognitive function remains to be elucidated.

Hirvonen, Laakso, et al. (2009) suggested that CC homozygosity is associated with higher dopamine availability in striatum and it has further been shown that psychosocial stress increases striatal dopamine levels in individuals reporting low early life parental care (Pruessner, Champagne, Meaney, & Dagher, 2004). As CC homozygotes have higher levels of dopamine available in the striatum, it may be hypothesized that stress‐induced increase in dopamine function is particularly detrimental to CC homozygotes, possibly leading to a hyperdopaminergic state in the subcortical areas. Furthermore, increased striatal dopamine levels are directly related to altered prefrontal activation in individuals at risk for psychosis (Fusar‐Poli et al., 2011) and in those with established schizophrenia (Meyer‐Lindenberg et al., 2002), but the effect on behavioral outcomes is not clear. Following on from the current findings, future studies would benefit from investigating the *DRD2* genotype and stress interactions on tests more specific to response inhibition.

There were several limitations in this study, the first of which being the sample size which prevented further analyses of potential interest such as investigating any interaction between the *DRD2* and *COMT* variants (Xu et al., 2007) and subgroup analysis of the trauma scale, given previous findings of the differential effects of trauma type on cognition (Green et al., 2014). Furthermore, we have no information about the mRNA or protein level expression of genes under investigation in the individuals who took part in this study, nor about other genetic polymorphisms that might affect aspects of the dopaminergic systems such as those encoding dopamine transporter, (Sesack, Hawrylak, Matus, Guido, & Levey, 1998), and monoamine oxidase A (*MAOA*) which was recently shown to interact with early life stress (Zohsel et al., 2015). *C957T* is also likely to be in linkage disequilibrium with *Taq1A* polymorphism of *ANKK1* gene (Frank & Hutchison, 2009). What is more, *DRD2* affects other aspects of behavior not investigated in this study, such as reward, addiction (Volkow, Fowler, Wang, & Swanson, 2004) and impulsivity (White, Lawford, Morris, & Young, 2009), suggesting that the effect of *C957T* on behavior and its interactions with other gene polymorphisms and environmental stressors are more complex than observed in this study. We also acknowledge that a subset of the participants may have been experiencing current adversity, and a small number of individuals (*n *=* *6) reported a history of mental health disorder, but these participants were not disproportionately distributed between genotype groups and it is therefore unlikely that these cases were driving the significant effects seen in our study. However, our sample comprised a male only population, predominately of Caucasian origin, which may have improved our ability to find variant effects specific to this population given mixed findings for general effects across gender and ethnicity for both *COMT* (Domschke, Deckert, O'Donovan, & Glatt, 2007; Gurvich & Rossell, 2015; Harrison & Tunbridge, 2008) and *DRD2* (Gurvich & Rossell, 2015; Liu et al., 2014; Villalba et al., 2015). However, a recent study by Gurvich and Rossell (2015) failed to find an effect of *COMT Val158Met* genotype on cognition in males and it has been proposed that higher estrogen levels in females might amplify the effects of *COMT*, therefore affecting dopamine function differently from males (Colzato & Hommel, 2014; Gurvich & Rossell, 2015). Further studies should investigate the effects of these genotypic variants and the interaction of stress in healthy females.

In summary, we provide evidence for the role of *DRD2 C957T* polymorphism in several executive function domains, namely working memory, executive planning and sustained attention. We have also provided evidence of the limited effect that the *COMT Val158Met* SNP has on executive function in healthy adult males and that this does not seem to be modulated by the occurrence of early life stress. Importantly, this is the first study to indicate that *DRD2 C975T* and early life stress interact to impact on executive function and future research should investigate this further. These findings provide support for the interaction of genetic vulnerability with stressful life events in contributing to cognitive functioning. Importantly, furthering our understanding of the fundamental cognitive processes inherently involved in disorders such as schizophrenia and the way in which genetic risk factors confer their biological vulnerability will enable us to disentangle the key components leading to the development of these conditions.

## Conflict of Interest

None declared.
