# Supplementary material for: The effect of COMT Val158Met and DRD2 C957T polymorphisms on executive function and the impact of early life stress
Source: Brain Behav. 2017 Apr 12;7(5):e00695. doi: 10.1002/brb3.695 (PMC5434197; doi:10.1002/brb3.695)
Supplement: Supplementary file 1 [file BRB3-7-e00695-s001.pptx]

## Slide 1
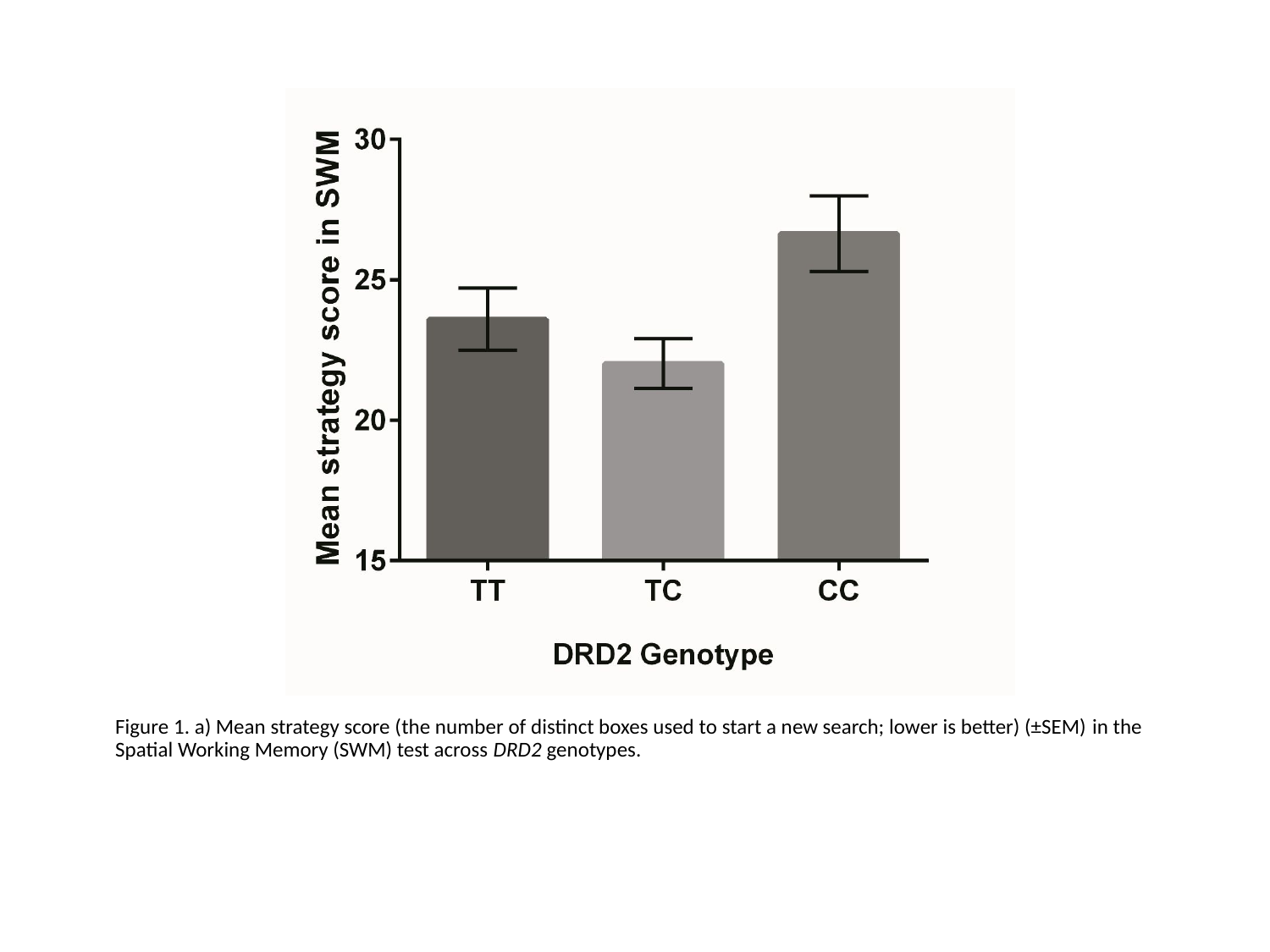

Figure 1. a) Mean strategy score (the number of distinct boxes used to start a new search; lower is better) (±SEM) in the Spatial Working Memory (SWM) test across DRD2 genotypes.

## Slide 2
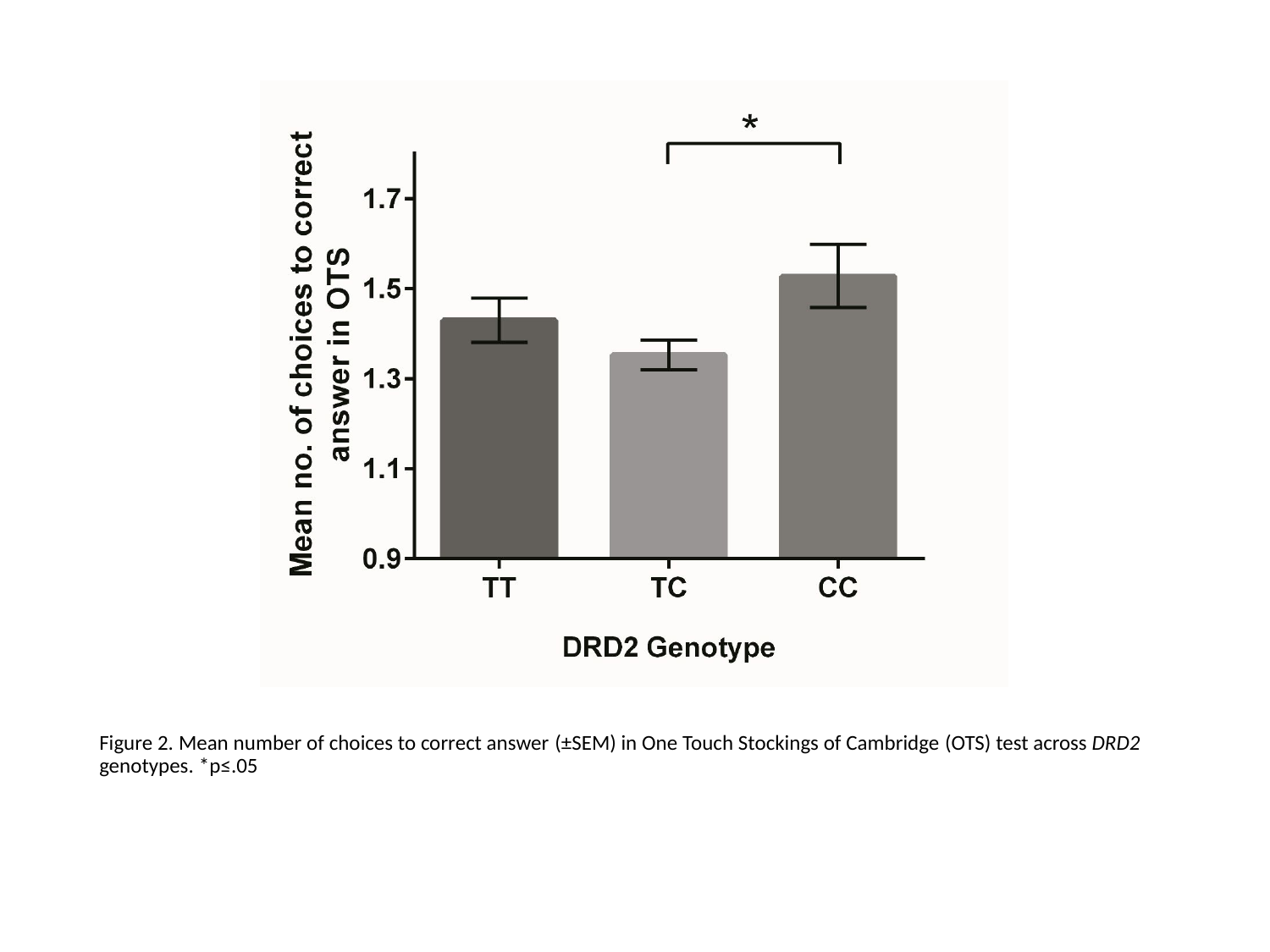

Figure 2. Mean number of choices to correct answer (±SEM) in One Touch Stockings of Cambridge (OTS) test across DRD2 genotypes. *p≤.05

## Slide 3
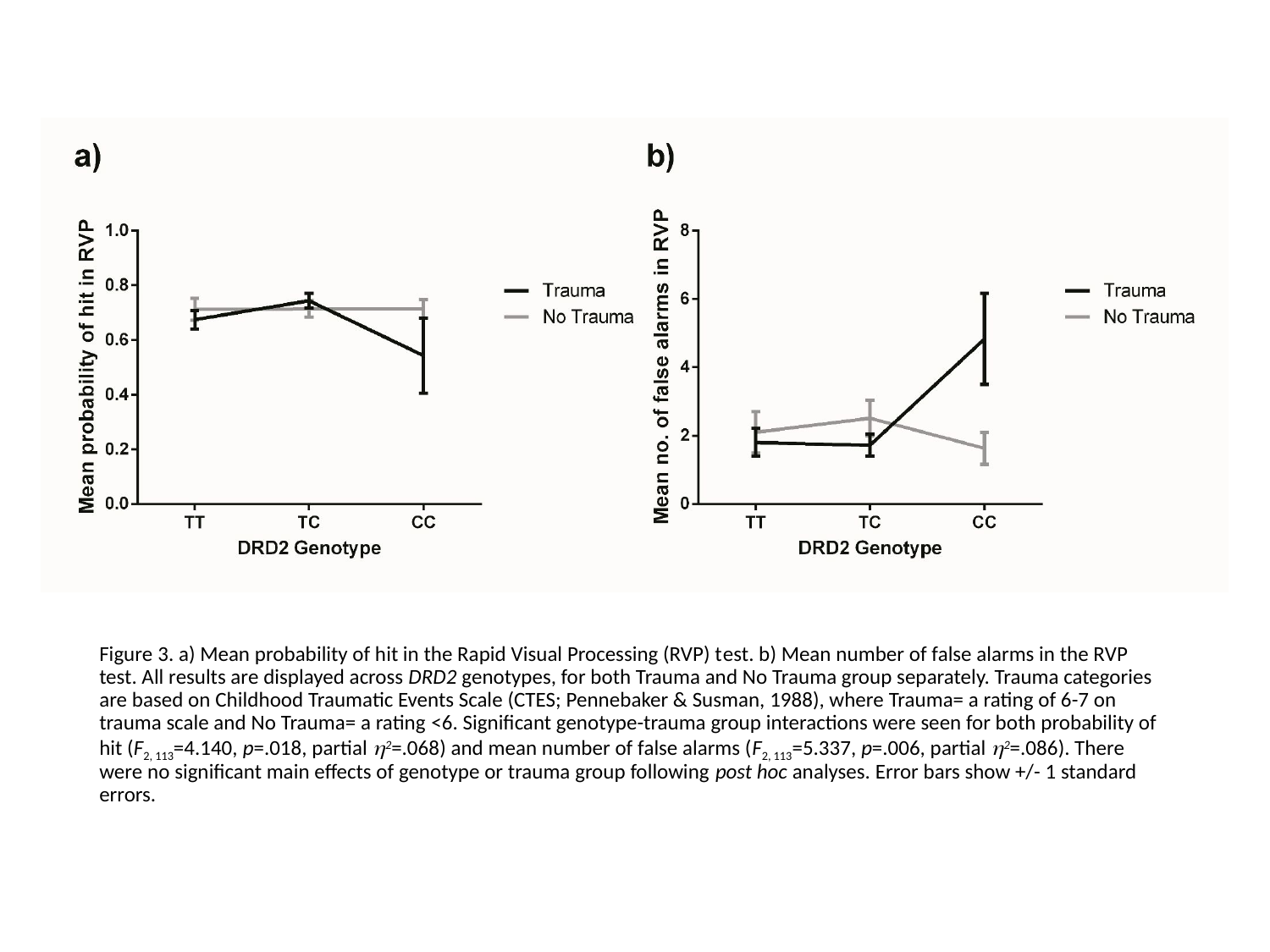

Figure 3. a) Mean probability of hit in the Rapid Visual Processing (RVP) test. b) Mean number of false alarms in the RVP test. All results are displayed across DRD2 genotypes, for both Trauma and No Trauma group separately. Trauma categories are based on Childhood Traumatic Events Scale (CTES; Pennebaker & Susman, 1988), where Trauma= a rating of 6-7 on trauma scale and No Trauma= a rating <6. Significant genotype-trauma group interactions were seen for both probability of hit (F2, 113=4.140, p=.018, partial 2=.068) and mean number of false alarms (F2, 113=5.337, p=.006, partial 2=.086). There were no significant main effects of genotype or trauma group following post hoc analyses. Error bars show +/- 1 standard errors.
